# Myofibroblastoma of the Breast: Literature Review and Case Report

**DOI:** 10.1155/2016/1714382

**Published:** 2016-07-25

**Authors:** Mario Metry, Mohamad Shaaban, Magdi Youssef, Michael Carr

**Affiliations:** Breast Surgery Unit, Northumbria Healthcare NHS Foundation Trust, Woodhorn Lane, Ashington NE63 9JJ, UK

## Abstract

Myofibroblastoma of the breast is a rare benign spindle cell tumor. The main aim of this study is to review the literature of this rare tumor. We present a case of a mammary myofibroblastoma occurring in an 82-year-old man, emphasizing the clinical, radiological, and pathological features. The tumor was successfully identified and managed in our hospital. We would like to draw the attention of clinicians to myofibroblastoma as a rare possibility in the differential diagnosis of a breast mass.

## 1. Introduction

Recently, it has been confirmed that mammary myofibroblastoma belongs to the category of the benign mesenchymal tumors showing deletion of 13q14 region, together with spindle cell lipoma and cellular angiofibroma [1].

Myofibroblastoma was first reported by Wargotz et al. in 1987, as a benign spindle cell tumor of the breast with myofibroblastic features [2]. Only a few cases of this tumor have been reported in the English literature, so that the report of a new case gave us the opportunity to review the clinical management of myofibroblastoma.

## 2. Case Presentation

An 82-year-old man presented to the low-risk breast clinic with a few days' history of a tender lump in his left breast. He gave no family history of breast or ovarian cancer and was a nonsmoker. He suffered from ischemic heart disease and was on medications for benign prostatic hyperplasia.

Physical examination revealed a 20 mm smooth, mobile mass E2, situated asymmetrically behind the left areola at the 11 o'clock position, towards the upper inner quadrant of the breast tissue. This was nontender and there was no associated axillary lymphadenopathy. In addition, there was mild, diffuse, clinically benign gynecomastia on the right breast.

**Figure 1:**
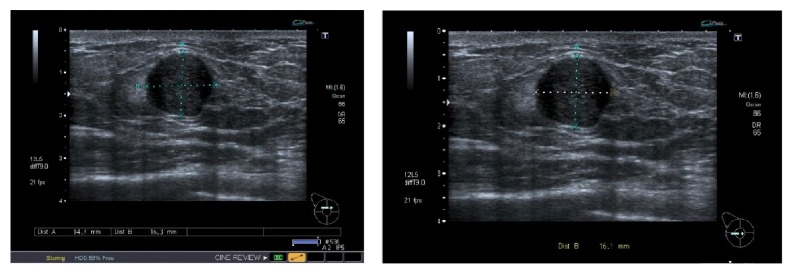
Ultrasound scan showing the lesion 15 × 16 mm mass (U3).

Ultrasound scan examination showed the symptomatic lesion of the left breast as a 16 × 15 mm rounded hypoechoic mass U3 (Figure 1). There was no evidence of gynecomastia.

A USS guided core biopsy was carried out from the mass. Histological examination confirmed a well-circumscribed mesenchymal lesion consisting of bland-looking spindle-shaped cells arranged in interlacing short bundles interrupted by keloidal-like, brightly eosinophilic collagen bands. No atypia or mitotic activity was seen.

Immunohistochemistry showed a positive reaction with alpha-smooth muscle actin (SMA), desmin, and CD34. Neoplastic cells were also positive for estrogen receptor (ER), but they were negative with MNF116, S100, and p63. Based on these morphological and immunohistochemical features, the diagnosis of “classic type myofibroblastoma of the breast was rendered”.

Options of treatment were discussed with the patient; the patient opted for excision of the mass.

Uneventful excision was performed from which the patient made a rapid and uncomplicated recovery.

Macroscopic examination revealed a circumscribed tumor mass measuring 15 mm in greatest diameter, with a specimen weight of 2.75 grams.

**Figure 2:**
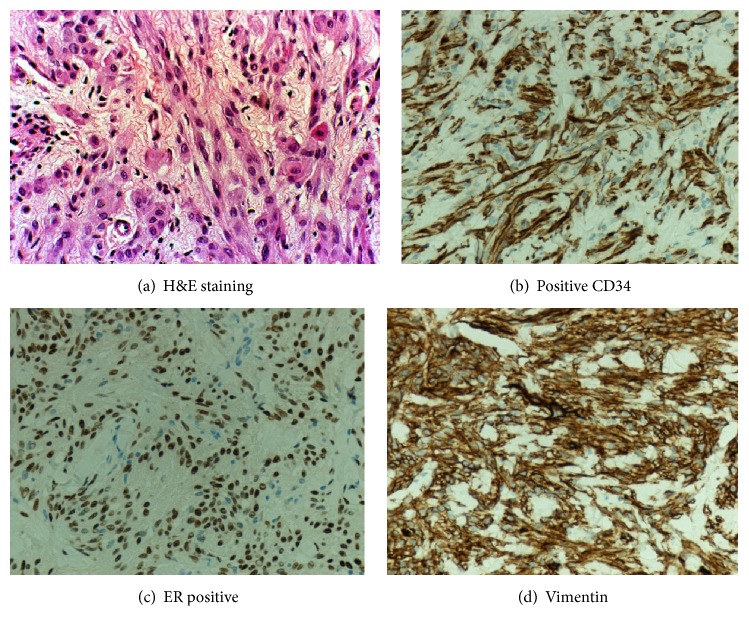
The histological pictures of the specimen as obtained from the histopathology department.

Histological examination showed a well-circumscribed mesenchymal tumor with features similar to those of the relative core biopsy. It consisted of short fascicles of spindle cells with pale cytoplasm and oval nuclei, with interspersed thick collagen bands. Although the tumor was moderately cellular, there was neither nuclear atypia nor mitoses (Figure 2(a)). Immunohistochemistry studies showed positive CD34 (Figure 2(b)), moderately positive for desmin, SMA, and ER (Figure 2(c)), and diffuse immunoreactivity for vimentin (Figure 2(d)). Pancytokeratin staining was negative, while CD31 highlighted intratumoral blood vessels.

## 3. Discussion and Literature Review

Myofibroblasts play an important role in the response to tissue injury. Damaged cells and some malignant tumor cells produce cytokines, particularly transforming growth factor *β*1, causing fibroblasts to migrate into the injured tissue. They begin to develop smooth muscle actin fibers, and they are transformed into myofibroblasts with contractile ability. Contraction of injured tissue speeds the processes of healing and repair [3].

Myofibroblastoma has recently been described as a rare benign mesenchymal tumor which usually occurs in the breast parenchyma of both females and males [4]. Most cases of myofibroblastoma occur most often in women and men aged 40–87 years. It tends to affect older men and postmenopausal women [5–8]. Characteristically, these lesions present as a solitary, painless, firm, and freely mobile mass which grows slowly for several months or years [8, 9]. It can exhibit a wide range of histological patterns including the following: collagenized/fibrous, cellular, lipomatous, infiltrative, myxoid, epithelioid, and deciduoid-like variant [10, 11].

Histologically, myofibroblastoma is composed of bipolar spindle-shaped cells arranged in short intersecting fascicles interrupted by keloidal-like eosinophilic collagen bands. Mammary ducts or lobules are characteristically absent. Macroscopically, the cut surface shows a well-demarcated pale pink or tan round mass [8–11]. Immunohistochemically, myofibroblastoma is positive for vimentin and CD34 and variably positive for desmin and SMA. It is also positive for CD10, CD99, estrogen, progesterone receptors, and bcl-2 protein and only focally positive for h-caldesmon. S100 protein, HMB-45, epithelial markers (EMA and pancytokeratins), and C-kit (CD117) are consistently negative. Immunohistochemical results are consistent with the fibroblastic/myofibroblastic nature of the neoplastic cells [1, 12–15]. Unlike mammary-type myofibroblastoma, myofibroblastoma that primarily arises in the lymph nodes exhibits nuclear palisading. Some reported cases represent a hitherto unreported variant of mammary-type myofibroblastoma closely mimicking schwannoma [16].

The appearances of myofibroblastoma on imaging are nonspecific. On sonography, it shows a homogeneously hypoechoic well-circumscribed solid mass which resembles fibroadenoma. The mammographic findings usually consist of a well-circumscribed round or oval dense and noncalcified mass [17]. The MRI finding (although not often done) shows a homogeneously enhanced mass with internal septations [18–22]. Most reported cases vary between 10 and 37 mm in size although much larger tumors have recently been described [23, 24].

Given the nonspecific radiological appearances, the final diagnosis of myofibroblastoma requires a needle core biopsy. Myofibroblastoma can be treated with local excision mainly for symptomatic relief; local recurrence is not a recognized feature of myofibroblastoma [8, 9].

## 4. Conclusion

Myofibroblastoma is a rare breast tumor occurring in both postmenopausal women and elderly men. Triple assessment by clinical examination, ultrasound scanning, and needle core biopsy will lead to an accurate diagnosis. Recurrence is unlikely following excision with clear resection margins.

We would like to draw the attention of clinicians to myofibroblastoma as a rare possibility in the differential diagnosis of a breast mass with well-circumscribed margins.

## References

[1] Magro G., Righi A., Casorzo L. (2012). Mammary and vaginal myofibroblastomas are genetically related lesions: fluorescence in situ hybridization analysis shows deletion of 13q14 region. *Human Pathology*.

[2] Wargotz E. S., Weiss S. W., Norris H. J. (1987). Myofibroblastoma of the breast: sixteen cases of a distinctive benign mesenchymal tumor. *American Journal of Surgical Pathology*.

[3] Magro G. (2009). Epithelioid-cell myofibroblastoma of the breast: expanding the morphologic spectrum. *The American Journal of Surgical Pathology*.

[4] Magro G. (2016). Mammary myofibroblastoma: an update with emphasis on the most diagnostically challenging variants. *Histology and Histopathology*.

[5] Magro G., Bisceglia M., Michal M., Eusebi V. (2002). Spindle cell lipoma-like tumor, solitary fibrous tumor and myofibroblastoma of the breast: a clinico-pathological analysis of 13 cases in favor of a unifying histogenetic concept. *Virchows Archiv*.

[6] Reis-Filho J. S., Faoro L. N., Gasparetto E. L., Totsugui J. T., Schmitt F. C. (2001). Mammary epithelioid myofibroblastoma arising in bilateral gynecomastia: case report with immunohistochemical profile. *International Journal of Surgical Pathology*.

[7] Magro G. (2008). Mammary myofibroblastoma: a tumor with a wide morphologic spectrum. *Archives of Pathology and Laboratory Medicine*.

[8] Magro G., Salvatorelli L., Spadola S., Angelico G. (2014). Mammary myofibroblastoma with extensive myxoedematous stromal changes: a potential diagnostic pitfall. *Pathology Research and Practice*.

[9] Magro G., Gurrera A., Bisceglia M. (2003). H-caldesmon expression in myofibroblastoma of the breast: evidence supporting the distinction from leiomyoma. *Histopathology*.

[10] Magro G., Fraggetta F., Torrisi A., Emmanuele C., Lanzafame S. (1999). Myofibroblastoma of the breast with hemangiopericytoma-like pattern and pleomorphic lipoma-like areas. Report of a case with diagnostic and histogenetic considerations. *Pathology Research and Practice*.

[11] Magro G., Michal M., Vasquez E., Bisceglia M. (2000). Lipomatous myofibroblastoma: a potential diagnostic pitfall in the spectrum of the spindle cell lesions of the breast. *Virchows Archiv*.

[12] Iglesias A., Arias M., Santiago P., Rodríguez M., Mañas J., Saborido C. (2007). Benign breast lesions that simulate malignancy: magnetic resonance imaging with radiologic-pathologic correlation. *Current Problems in Diagnostic Radiology*.

[13] Magro G., Caltabiano R., Di Cataldo A., Puzzo L. (2007). CD10 is expressed by mammary myofibroblastoma and spindle cell lipoma of soft tissue: an additional evidence of their histogenetic linking. *Virchows Archiv*.

[14] Pina L., Apesteguía L., Cojo R. (1997). Myofibroblastoma of male breast: report of three cases and review of the literature. *European Radiology*.

[15] Hinz B., Phan S. H., Thannickal V. J., Galli A., Bochaton-Piallat M.-L., Gabbiani G. (2007). The myofibroblast: one function, multiple origins. *The American Journal of Pathology*.

[16] Magro G., Foschini M. P., Eusebi V. (2013). Palisaded myofibroblastoma of the breast: a tumor closely mimicking schwannoma: report of 2 cases. *Human Pathology*.

[17] Greenberg J. S., Kaplan S. S., Grady C. (1998). Myofibroblastoma of the breast in women: imaging appearances. *American Journal of Roentgenology*.

[18] Magro G., Michal M., Bisceglia M. (2001). Benign spindle cell tumors of the mammary stroma: diagnostic criteria, classification, and histogenesis. *Pathology Research and Practice*.

[19] Magro G., Amico P., Gurrera A. (2007). Myxoid myofibroblastoma of the breast with atypical cells: a potential diagnostic pitfall. *Virchows Archiv*.

[20] Dockery W. D., Singh H. R., Wilentz R. E. (2001). Myofibroblastoma of the male breast: imaging appearance and ultrasound-guided core biopsy diagnosis. *The Breast Journal*.

[21] Magro G., Bisceglia M., Michal M. (2000). Expression of steroid hormone receptors, their regulated proteins, and bcl-2 protein in myofibroblastoma of the breast. *Histopathology*.

[22] Magro G., Vecchio G. M., Michal M., Eusebi V. (2013). Atypical epithelioid cell myofibroblastoma of the breast with multinodular growth pattern: a potential pitfall of malignancy. *Pathology Research and Practice*.

[23] Tavassoli F. A., Devilee P. (2003). *WHO Classification of Tumors. Pathology and Genetics of Tumors of the Breast and Female Genital Body*.

[24] Magro G., Longo F. R., Salvatorelli L., Vasquez E., Vecchio G. M. (2014). Lipomatous myofibroblastoma of the breast: case report with diagnostic and histogenetic considerations. *Pathologica*.

